# Itraconazole inhibits the Wnt/β-catenin signaling pathway to induce tumor-associated macrophages polarization to enhance the efficacy of immunotherapy in endometrial cancer

**DOI:** 10.3389/fonc.2025.1590095

**Published:** 2025-07-08

**Authors:** Xin Guan, Lu Han

**Affiliations:** ^1^ Department of Graduate, Dalian Medical University, Dalian, Liaoning, China; ^2^ Department of Gynecology, The Third Affiliated Hospital of Dalian University of Technology, Dalian, Liaoning, China; ^3^ Department of Gynecology, Dalian Women and Children’s Medical Group, Dalian, Liaoning, China; ^4^ Laboratory of Early Diagnosis and Biotherapy of Malignant Tumors in Children and Women in Liaoning Province, Dalian Women and Children’s Medical Group, Dalian, Liaoning, China

**Keywords:** endometrial cancer, itraconazole, immune checkpoint inhibitors, tumor-associated macrophages, Wnt/β-catenin

## Abstract

**Background:**

Endometrial cancer (EC) is a common gynecologic malignancy with limited treatment options. This study aimed to evaluate the potential of itraconazole (ITZ), a widely used antifungal drug, as an anti-tumor agent and an adjuvant to immunotherapy for EC.

**Methods:**

The effects of ITZ on Ishikawa cells were assessed using proliferation assays, apoptosis assays, and invasion assays. The combination of ITZ and immune checkpoint inhibitors (ICIs) was evaluated to determine their synergistic effects on tumor invasion. Tumor-associated macrophages (TAMs) polarization and cytokine levels were analyzed by flow cytometry and enzyme linked immunosorbent assay (ELISA). Western blotting and Real-time reverse transcription polymerase chain reaction (RT-PCR) were used to investigate the impact of ITZ on the Wnt/β-catenin signaling pathway. Finally, *in vivo* experiments were conducted using a mice tumor model to validate the anti-tumor effects of ITZ and its combination with ICIs.

**Results:**

ITZ inhibits Ishikawa cells proliferation and invasion through apoptosis induction. When combined with ICIs, ITZ significantly enhanced the inhibition of tumor invasion, an effect associated with TAMs polarization. ITZ increased IFN-γ secretion, reduced IL-10 levels, and promoted TAMs polarization from the M2 to the M1 phenotype. Mechanistically, ITZ downregulated Wnt-3a and β-catenin expression while upregulating Axin-1, thereby suppressing Wnt/β-catenin signaling in TAMs. *In vivo*, ITZ and ICIs synergistically reduced tumor volume and weight, shifted TAMs polarization toward the M1 phenotype, and suppressed Wnt/β-catenin signaling.

**Conclusions:**

ITZ demonstrated robust anti-tumor activity against EC by inhibiting Ishikawa cells proliferation, invasion, and enhancing the efficacy of ICIs. Through its dual role in directly targeting tumor cells and modulating the tumor microenvironment, ITZ shows promise as a multitargeted therapeutic agent and a valuable adjuvant to immunotherapy for EC.

## Introduction

Endometrial cancer (EC), a prevalent gynecologic malignancy, was responsible for approximately 420,300 new cases and 97,800 deaths globally in 2022, representing 8.4% of new cancer cases and 1.7% of cancer-related deaths worldwide, respectively (1). Surgical intervention remains the primary treatment modality. However, adjuvant therapies such as radiotherapy and chemotherapy are considered in certain cases (2). While these adjuvant treatments can extend patient survival, they often significantly impair quality of life due to their associated toxicity and side effects, including myelosuppression, nausea, and vomiting (2–4). Furthermore, combining immunotherapy with other therapeutic approaches is also considered as a promising strategy (5). Recently, immunotherapy has demonstrated considerable potential in the management of various cancers, including EC. Research indicates that immune checkpoint inhibitors (ICIs), such as PD-1 and PD-L1 inhibitors, exhibit significant effectiveness in treating advanced EC, particularly in patients with mismatch repair deficiency (dMMR) (6, 7). Moreover, the integration of immunotherapy with additional therapeutic modalities is regarded as a promising strategy. Specifically, the combination of nivolumab and cabozantinib has demonstrated a significant enhancement in progression-free survival (PFS) among patients with EC, providing renewed hope for individuals who have not responded adequately to monotherapy with immunotherapy (7).

Tumor-associated macrophages (TAMs), as a critical element of the tumor microenvironment, are pivotal in influencing the immunotherapeutic efficacy against malignant tumors (8–10). Research has demonstrated that TAMs facilitate tumor cells proliferation and metastasis through the secretion of various cytokines, concurrently inhibiting anti-tumor immune responses (11, 12). Furthermore, the Wnt/β-catenin signaling pathway is implicated in the promotion of M2-type TAMs, which subsequently attenuates the efficacy of immunotherapy. Enhancing immunotherapeutic outcomes can be achieved by repolarizing TAMs, thereby converting them into M1-type TAMs that exhibit anti-tumor activity (13, 14). Inhibiting the Wnt/β-catenin signaling pathway represents a crucial approach for enhancing the functionality of TAMs and improving the immune response in patients with EC (8).

Itraconazole (ITZ), an azole antifungal medication, has been employed in the treatment of fungal infections for the past three decades, with its safety and efficacy well-established (15). Research on various malignancies, including hepatocellular carcinoma (16), non-small cell lung cancer (17), prostate cancer (18, 19), basal cell carcinoma (20), and glioblastoma (21) has demonstrated that ITZ effectively inhibits cell proliferation and invasion through apoptosis induction, and exhibits significant antitumor activity. Liang et al. (22) demonstrated that ITZ effectively inhibited melanoma growth and significantly extended survival in the mouse xenograft model. The study observed a marked down-regulation of Wnt-3a and β-catenin, which are pivotal components of the Wnt/β-catenin signaling pathway, alongside a significant up-regulation of Axin-1 following treatment. Furthermore, previous studies (16–21) on various malignancies have similarly reported that ITZ exerts antitumor effects by modulating the Wnt/β-catenin signaling pathway, specifically through the alteration of Wnt-3a, β-catenin, and Axin-1 expression.

Given the key role of TAMs in shaping the immune landscape of the tumor microenvironment and their regulation by the Wnt/β-catenin signaling pathway, we hypothesize that ITZ not only directly targets tumor cells to exert antitumor effects, but may also promote the conversion of TAMs to the pro-inflammatory M1 phenotype by inhibiting the Wnt/β-catenin signaling pathway, thereby enhancing the efficacy of immunotherapy. This study may provide new insights into the potential role of ITZ as an adjuvant in immunotherapy and offer a promising strategy to improve treatment outcomes in EC patients.

## Materials and methods

### Chemicals and materials

The reagents used in this study were as follows: Dulbecco’s modified eagle medium (DMEM) (SH30022.01, Cytiva, Shanghai, China), Roswell Park Memorial Institute (RPMI) 1640 medium (SH30096.01, Cytiva, Shanghai, China), fetal bovine serum (FBS) (KY-01000S, Kangyuan, Anhui, China), penicillin–streptomycin (Pen-Strep) (PS2004HY, Haoyang, Tianjin, China), trypsin (T1320, Solarbio, Beijing, China), phosphate buffered saline (PBS) (BL302A, Biosharp, Anhui, China), dimethyl sulfoxide (DMSO) were from Sigma-Aldrich (St. Louis, MO, USA). Itraconazole (ITZ, I8250, Solarbio, Beijing, China), PD-1/PD-L1 inhibitor 2 (as ICI, BMS202, Selleck, Shanghai, China).

The primary antibodies against wingless-type MMTV integration site family (Wnt-3a, A13601, 1:800), axis inhibition protein 1 (Axin-1, A16019, 1:500), beta-catenin (β-catenin, A19657, 1:500) were purchased from ABclonal (Wuhan, China). The primary antibodies against glyceraldehyde-3-phosphate dehydrogenase (GADPH, 10494-1-AP, 1:3000), CD8 (66868-1-Ig, 1:5000), CD56 (14255-1-AP, 1:2000), CD86 (26903-1-AP, 1:400), CD206 (60143-1-Ig, 1:2000), interleukin-10 (IL-10, 60269-1-Ig, 1:200), interferon-gamma (IFN-γ, 15365-1-AP, 1:200) and Ki-67 (27309-1-AP, 1:800) were purchased from Proteintech (Beijing, China).

### Cell line and cell culture

The Ishikawa, Hec50co, ECC-1, KLE, and RL95–2 cell lines were obtained from the American Type Culture Collection (Manassas, VA, USA). Ishikawa and Hec50co cells were cultured in Dulbecco’s Modified Eagle Medium (DMEM) supplemented with 10% fetal bovine serum (FBS) and 1% antibiotic (Pen-Strep) (Thermo, MA, USA). KLE cells were cultured in RPMI 1640 (Thermo, MA, USA) supplemented with 10% FBS and 1% Pen-Strep. ECC-1 cells were cultured in RPMI 1640 supplemented with 5% FBS and 1% Pen-Strep. RL95–2 cells were cultured in DMEM/F12 (Thermo, MA, USA) supplemented with 10% FBS and 1% Pen-Strep. All cell lines were maintained at 37°C in a humidified atmosphere with 95% O_2_ and 5% CO_2_.

CD8^+^ T cells was obtained from QINGQI Biotechnology Development Co., Ltd. (BFN60810741, Shanghai, China) and cultured with DMEM supplemented with 10% FBS and 1% Pen-Strep at 37°C in a humidified atmosphere with 95% O_2_ and 5% CO_2_. NK-92 cells was procured from iCell Bioscience (Shanghai, China) and cultured with Minimum Essential Medium (MEM) supplemented with 12.5% FBS and 12.5% horse serum at 37°C in a humidified atmosphere with 95% O_2_ and 5% CO_2_ (23, 24). To induce differentiation into tumor-associated macrophages (TAMs), THP-1 cells (CBP60518, Jiangsu, China) were seeded into 6-well plates at a density of 1 × 10^6^ cells/well in 2 mL of complete medium (RPMI 1640 medium containing 10% FBS and 1% Pen-Strep) at 37°C in a humidified atmosphere with 95% O_2_ and 5% CO_2_. Phorbol 12-myristate 13-acetate (PMA, S1819, Beyotime, Shanghai, China) was added to the medium at a final concentration of 100 nM, and the cells were incubated for 48 h. After induction, the medium containing PMA was removed, and cells were washed twice with PBS to remove non-adherent cells and residual PMA.

A cell co-culture system was constructed using a co-culture insert chamber (0.4 μm, Millipore, Merck, Germany) following the study of Gołąbek-Grenda et al. (25). Tumor cells (5 × 10^4^) and immune cells (5 × 10^4^) were inoculated in the upper and lower chambers, respectively, in a 1:1 ratio. The cells were filled with 700 μL of medium and incubated for 24 h at 37°C in a humidified atmosphere with 95% O_2_ and 5% CO_2_. For subsequent experiments, cells in different chambers were selected for analysis as needed.

Cytotoxicity studies

Five different cell lines (Ishikawa, Hec50co, ECC-1, KLE, and RL95-2) were pre-screened to identify the most suitable one for further study. The cell suspensions in the logarithmic growth phase were seeded into 96-well plates with 100 μL per well. Three parallel wells were used for each sample. The cells were treated with 50 μL of 5% DMSO and a range of concentrations of ITZ (5, 10, and 15 μM) for 24 h at 37°C.

In subsequent experiments, the Ishikawa cell line with better activity was selected. ITZ was set to the following concentrations: 5, 10, and 15 μM. Ishikawa cells suspensions in the logarithmic growth phase were inoculated into 96-well plates (8 × 103 cells/well) with 100 μL in each well. Three parallel wells were used in each sample. Ishikawa cells were treated with 50 μL of 5% DMSO and a range of concentrations of ITZ for 24 h, 48 h and 72 h at 37°C, respectively.

Cell viability was determined using the cell counting kit-8 (CCK-8, C0038, Beyotime, Shanghai, China). After treatment, the old medium was discarded. Then, 10 μL of CCK-8 reagent was added to each well, and the optical density values were measured at a wavelength of 450 nm using an enzyme marker (K3 PLUS, BIO-DL, Shanghai, China) after incubation at 37°C for 1 h. All experiments were performed in triplicate, and the data were averaged across three independent experiments.

### Cell apoptosis assay

The Annexin V-FITC cell apoptosis assay kit (C1062S, Beyotime, Shanghai, China) was used to measure the proportion of cells undergoing apoptosis. The Ishikawa cells suspension in the logarithmic growth phase was inoculated into 6-well plates at a density of 1 × 10^6^ cells per well. Three parallel wells were used per sample. Ishikawa cells were treated with 50 μL 5% DMSO or 5, 10 or 15 μM ITZ at 37°C for 24 h. Subsequently, the cells were washed with phosphate-buffered saline (PBS, pH = 7.4), centrifuged at 1000 r/min for 5 min, and then resuspended. A volume of 5 µL of staining solution was added, and the cells were incubated at 37°C for 30 min in the dark. The intensity of red fluorescence was measured using a FACSVerse™ flow cytometer (BD Biosciences, Franklin Lakes, NJ, USA) with an excitation wavelength of 450 nm. The cell apoptosis was quantified using ModFit LT 5.0 software (Cytonome Verity, Bedford, MA, USA).

### Invasion assay

**Figure 1:**
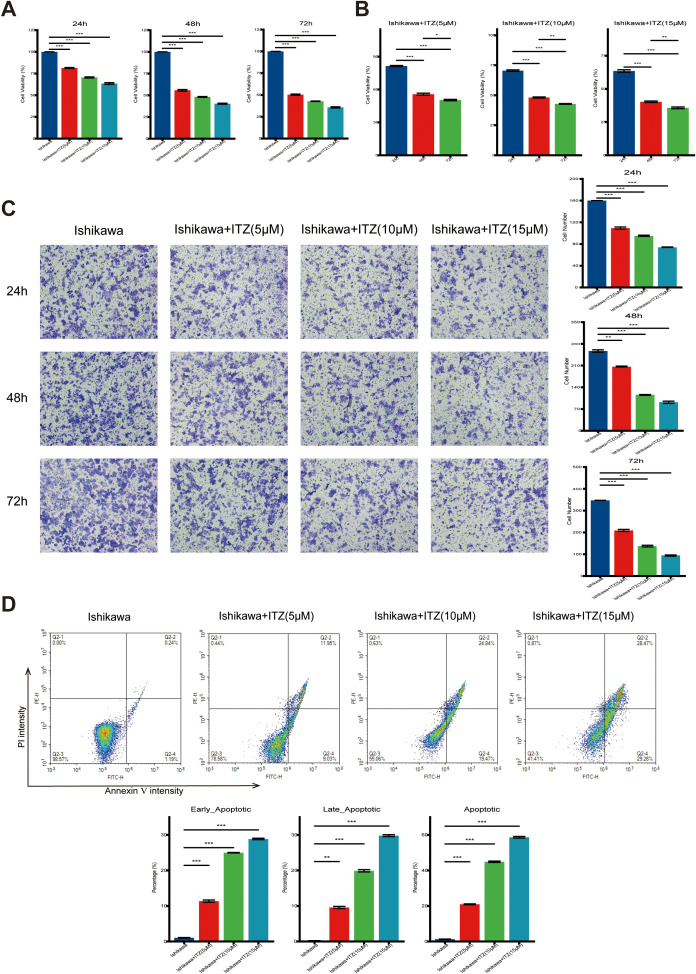
ITZ inhibits cell proliferation and promotes apoptosis in Ishikawa cells. **(A-B)** The viability of Ishikawa cells treated with increasing concentrations of ITZ (5 µM, 10 µM, and 15 µM) for 24, 48, and 72 h was assessed using the CCK-8 assay. **(C)** Transwell invasion assay showing the invasive ability of Ishikawa cells treated with ITZ (5 µM, 10 µM, and 15 µM) for 24, 48, and 72 h. **(D)** Apoptosis of Ishikawa cells treated with ITZ at various concentrations was evaluated by flow cytometry. *P < 0.05, **P ≤ 0.01, ***P ≤ 0.001.

Invasion assay was performed using 24-well plates. For the experiment, the inserts were pre-warmed with cell culture medium, PBS, and trypsin at 37°C. Logarithmic phase Ishikawa cells were harvested, prepared as single-cell suspensions, and seeded into the upper chambers at a density of 5 × 10^4^ cells in 200 μL serum-free medium. The lower chambers were filled with 600 μL of medium containing various drugs and co-cultured cells, as follows: *Ishikawa*: 600 μL medium; *Ishikawa+ITZ*: 10 μM ITZ; *Ishikawa+TAMs*: 200 μL TAMs; *Ishikawa+TAMs+ITZ*: 10 μM ITZ + 200 μL TAMs; *Ishikawa+TAMs+Anti_PD-1*: 2 μM BSM202 + 200 μL TAMs; *Ishikawa+TAMs+ITZ+Anti_PD-1*: 10 μM ITZ + 2 μM BSM202 + 200 μL TAMs. The schematic illustration of the treatment regimen to clarify dosing and agent selection, please refer to **Figure 1A**.

After incubation at 37°C, non-migratory cells on the upper surface of the inserts were removed using cotton swabs. The inserts were fixed with ice-cold methanol for 20 min and stained with 0.1% crystal violet for 30 min. Excess stain was washed away with PBS, and migrated cells on the underside of the inserts were imaged under a light microscope at 24, 48 and 72 h. Three fields per well were captured for analysis.

### Flow cytometry analysis of TAMs phenotype

TAMs cells were harvested and washed twice with PBS. Approximately 1 × 10^6^ cells were resuspended in 100 μL of PBS and incubated at room temperature for 30 min. Afterward, 4 μL of CD206 antibody (FITC-65155, Proteintech, Hunan, China) and 5 μL of CD86 antibody (PE-65165, Proteintech, Hunan, China) were added, and the mixture was incubated in the dark at 4°C for 40 min. Following incubation, cells were washed once with PBS, and the supernatant was discarded. Next, cells were re-suspended in 500 μL of PBS and thoroughly mixed. Flow cytometry analysis was performed using NovoCyte Flow Cytometer (D1041, ACEA, Beijing, China) and NovoExpress analysis software (v.1.2.5)

### Enzyme-linked immunosorbent assay

The concentrations of interleukin-10 (IL-10) and interferon-gamma (IFN-γ) in the culture supernatants of TAMs were determined using commercial ELISA kits (IL-10: PI528; IFN-γ: PI511; Beyotime, Shanghai, China), following the manufacturer’s protocols. Briefly, cell culture supernatants were collected and centrifuged for 5 min to remove debris. ELISA plates pre-coated with capture antibodies were incubated with 100 μL of standards or samples per well at room temperature for 2 h. After washing five times, 100 μL of biotinylated detection antibodies was added to each well and incubated for 1 h, followed by incubation with 100 μL of horseradish peroxidase (HRP)-conjugated streptavidin for 20 min in the dark. Subsequently, 100 μL of TMB substrate solution was added and the plates were incubated in the dark for another 20 min. The reaction was stopped by adding 50 μL of stop solution to each well, and absorbance was measured at 450 nm using a microplate reader. Cytokine concentrations were calculated based on standard curves generated in parallel, and results were expressed in pg/mL.

### Real-time reverse transcription polymerase chain reaction

The TAMs were collected. Total RNA was extracted using the RNAex Pro reagent (AG21102, Agbio, Hunan, China). RT-PCR was conducted using ABScript III RT Master Mix for qPCR with gDNA Remover (RK20429, ABclonal, Wuhan, China). cDNA synthesis was quantified using 2X Universal SYBR Green Fast qPCR Mix (RK21203, ABclonal, Wuhan, China) and ABI Prism 7500 Sequence Detection System (Thermo Fisher, Waltham, MA, USA). The amplification regime was conducted with 40 cycles of 95°C for 15 s, 61°C for 15 s, and 72°C for 20 s. GADPH was used as an internal control. The primer sequences are shown in **Table 1**.

**Table 1:** The sequence of the primers.

Gene	Primer sequence (forward)	Primer sequence (reward)
Wnt-3a	CTCCTCTCGGATACCTCTTAGTG	GCATGATCTCCACGTAGTTCCTG
Axin-1	GATGAGGACGATGGCAGAGA	AATAGGGGTTGACTGGCTCC
β-catenin	TTCAGCAGAAGGTCCGAGTG	ATCACCACGTCCTCTGCAC
GAPDH	AACTCAGGAGAGTGTTTCCTCG	TGCCGTGAGTGGAGTCATAC

Wnt-3a, wingless-type MMTV integration site family member 3a; Axin-1, axis inhibition protein 1; β-catenin, beta-catenin; GAPDH, glyceraldehyde-3-phosphate dehydrogenase.

### Western blotting

The TAMs or fresh tumor tissues were collected. Protein lysates were prepared from 2 × 10^6^ cells or 20 mg of tissue using lysis buffer supplemented with protease and phosphatase inhibitors (200:1 ratio, freshly prepared). The samples were homogenized and incubated on ice for 30 min, followed by centrifugation at 12,000 rpm for 20 min at 4°C. The supernatants were collected, and protein concentrations were determined using a BCA protein quantification kit (abs9232, Absin, Shanghai, China) according to the manufacturer’s protocol. Equal amounts of protein (30 μg) were mixed with 5× loading buffer, boiled at 100°C for 10 mi, and separated on 10-15% SDS-polyacrylamide gels (PG112, Epizyme, Shanghai, China) by electrophoresis. Proteins were then transferred onto polyvinylidene difluoride (PVDF) membranes, which had been pre-activated with methanol and equilibrated in transfer buffer.

Membranes were blocked with 5% skimmed milk in TBST (tris-buffered saline with 0.1% Tween 20) for 1 h at room temperature, then incubated overnight at 4°C with primary antibodies diluted in TBST. After washing three times with TBST, the membranes were incubated with horseradish peroxidase (HRP)-conjugated secondary antibodies for 1 h at room temperature. Signal detection was performed using a chemiluminescent substrate (180-501, Tanon, Shanghai, China) and visualized using a chemiluminescent imaging system. Protein bands were quantified using ImageJ software (v1.52).

### Animals housing

The experimental mice were BALB/c nude mice (SPF grade, male, 4 weeks old, n = 24) purchased from the Silaike Jingda Laboratory Animal Co., Ltd. (No.430727231100089172, Hunan, China). All mice were housed under specific pathogen-free conditions, with a controlled temperature of 22–26 °C, relative humidity of 50–60%, and a 12h light/dark cycle. The mice were provided with sterilized food and water ad libitum. Before the experiments, all mice underwent acclimatization for 1 week. The study was authorized and reviewed by the Third Affiliated Hospital of Dalian University of Technology Ethics Committee (2024–185–001).

### Tumor xenograft model and tumor administration in nude mice

**Figure 2:**
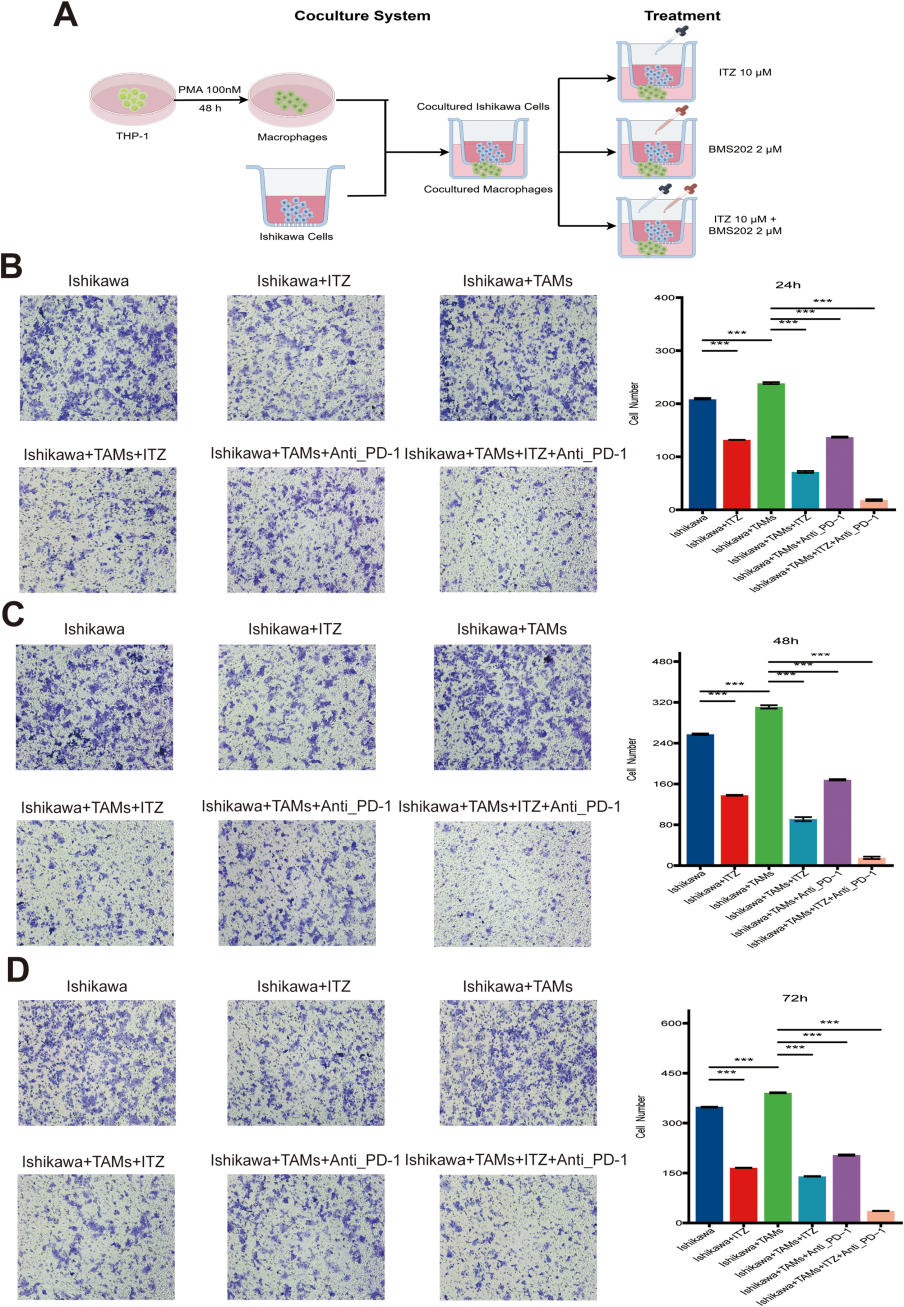
ITZ synergizes with ICI to suppress the invasion of Ishikawa cells. **(A)** The schematic illustration of the treatment regimen to clarify dosing and agent selection. **(B-D)** Transwell invasion assay showing the invasive ability of Ishikawa cells treated with ITZ, TAMs, and ICI individually or in combination for 24, 48, and 72h. **P ≤ 0.01, ***P ≤ 0.001.

Female BALB/c nude mice (4 weeks old) were first subjected to adaptive feeding for 7 days to allow acclimatization to the laboratory environment. On day 7, mice were subcutaneously inoculated with 3 × 10^6^ Ishikawa cells suspended in 100 μL PBS in the right flank. Immediately following tumor cells inoculation, treatment regimens were initiated in parallel, as outlined below: *Control*: Mice received intragastric administration (i.g.) of 1 mL PBS twice daily (bid) for 28 days, and 1 mL PBS by intraperitoneal injection (i.p.) on days 7, 13, and 20; *ITZ*: intragastric administration (i.g.) of 30 mg/kg ITZ twice daily (bid) for 28 days, and 1 mL PBS by intraperitoneal injection (i.p.) on days 7, 13, and 20; *Anti_PD-1*: intragastric administration (i.g.) of 1 mL PBS twice daily (bid) for 28 days, and 20 mg/kg BMS202 by intraperitoneal injection (i.p.) on days 7, 13, and 20; *ITZ+Anti_PD-1*: intragastric administration (i.g.) of 30 mg/kg ITZ twice daily (bid) for 28 days, and 20 mg/kg BMS202 by intraperitoneal injection (i.p.) on days 7, 13, and 20. Tumor growth was monitored every five days from the day of inoculation (day 7) until euthanasia on day 37. Tumor size was measured using a digital caliper, and volume was calculated with the formula: *V* = 0.52 × *L* × *W* ^2^. After the conclusion of the treatment (day 35), the animals’ mobility, grooming, and overall health were closely observed to ensure the well-being of the mice during the study period. At the end of the experiment, all mice were euthanized using CO_2_ asphyxiation in accordance with the 2020 edition of the American Veterinary Medical Association Guidelines for the Euthanasia of Animals, and tumor tissues were collected for further analysis. For a detailed experimental timeline and treatment schedule, please refer to **Figure 2A**.

### Immunohistochemistry

Tissue sections (4 μm) were prepared, deparaffinized, and rehydrated through graded ethanol. Antigen retrieval was performed using heat-induced method. Endogenous peroxidase activity was blocked with 3% hydrogen peroxide for 15 min. Sections were incubated overnight at 4°C with primary antibody: CD8 (66868-1-Ig, Proteintech, Wuhan, China); CD56 (14255-1-AP, Proteintech, Wuhan, China); CD86 (28058-1-AP, Proteintech, Wuhan, China); CD206 (60143-1-Ig, Proteintech, Wuhan, China); IL-10 (60269-1-Ig, Proteintech, Wuhan, China); IFN-γ (15365-1-AP, Proteintech, Wuhan, China); Ki-67 (27309-1-AP, Proteintech, Wuhan, China). The sections were washed with PBS and incubated with an HRP-conjugated secondary antibody (SP-9000, Zsbio, Beijing, China) for 20 min at room temperature. Visualisation was performed using 3, 3’-diaminobenzidine tetrahydrochloride (DAB, AR1021, Boster, Wuhan, China). Counterstaining was performed with hematoxylin. After dehydration and clearing, the slides were mounted and scanned using a whole-slide imaging system (SQS-40R, Shengqiang, Shenzhen, China).

### Statistical analysis

Data are expressed as the mean ± standard error of the mean. The cell experiments were conducted with least three replicates in each group. Parametric tests (Student’s t-test or one-way ANOVA analysis) were used to assess differences between groups for data that met both normal distribution and chi-square, otherwise, non-parametric tests (Wilcoxon test or Kruskal–Wallis test) were used, followed by Tukey’s *post hoc* test. *P < 0.05* was considered statistically significant

## Results

### Itraconazole inhibits Ishikawa cells proliferation and invasion through apoptosis induction

It was found that following 24 h of ITZ treatment (5 µM, 10 µM, and 15 µM), the cell survival rate of all five cell lines (Ishikawa, Hec50co, ECC-1, KLE, and RL95-2) was significantly reduced (**Supplementary Figure S1A**) (all *P ≤ 0.01*). Furthermore, this inhibitory effect in Ishikawa and Hec50co increased with higher ITZ concentrations, while other cell lines did not exhibit this trend (**Supplementary Figure S1A**). Moreover, at an ITZ concentration of 10 μM, the cell viability of the Hec50co cell line was found to be approximately 30% (**Supplementary Figure S1A**). Following a comprehensive consideration of the requirements for cell viability in subsequent experiments, it was determined that the Ishikawa cell line would be the optimal subject for further study.

The cell viability was markedly reduced following treatment with increasing concentrations of ITZ (5 µM, 10 µM, and 15 µM) for 24, 48, and 72 h (**Figures 1A, B**). Notably, the inhibitory effect became more pronounced with higher ITZ concentrations (15 µM vs. 10 µM or 5 µM) (**Figure 1A**) and with extended exposure times (72 h vs. 48 h or 24 h) (**Figure 1B**). Statistical analysis confirmed that the reduction in cell viability was highly significant across different concentrations and time points (all *P ≤ 0.001*), indicating a robust dose- and time-dependent anti-proliferative effect of ITZ on Ishikawa cells. In order to investigate the effects of ITZ on Ishikawa cells invasion, an invasion assay was performed for 24, 48 and 72 h at different concentrations. The results demonstrated that ITZ treatment significantly inhibited the invasive capacity of Ishikawa cells at all time points (all *P ≤ 0.01*), with the inhibitory effect becoming more pronounced as the treatment concentration increased (**Figure 1C**).

Moreover, ITZ was found to promote apoptosis in Ishikawa cells (**Figure 1D**). Results showed an increased percentage of apoptotic cells with rising ITZ concentrations. Quantitative analysis revealed a significant increase in both early and late apoptotic populations with ITZ treatment compared to untreated cells (all *P ≤ 0.01*). At the highest concentration (15 µM), ITZ induced a notable shift, with early apoptotic cells comprising approximately 30% and total apoptotic cells exceeding 60% of the population (**Figure 1D**). The present findings suggest that inhibits Ishikawa cells proliferation and invasion through apoptosis induction.

### Itraconazole synergizes with immune checkpoint inhibitors to inhibit Ishikawa cells invasion

To further investigate the potential effects of ITZ on immune cells and to ascertain whether it contributes to the therapeutic effects of ITZ on Ishikawa cells, co-culture systems were established between Ishikawa cells and CD8^+^ T, NK, and TAMs. The schematic diagram of the TAM co-culture system is shown in **Figure 2A**, with the remaining co-culture systems similar. The cells were exposed to 10 μM ITZ for 24 h. Subsequently, Ishikawa cells were extracted for the purpose of invasion assays. The results demonstrated that treatment with CD8^+^ T and NK cells significantly attenuated the invasive capacity of Ishikawa cells (*all P ≤ 0.001*) (**Supplementary Figure S1B**), while treatment with TAMs markedly enhanced the invasive capacity of Ishikawa cells (*all P ≤ 0.001*) (**Figures 2B–D**). Furthermore, when ITZ was used in combination with CD8^+^ T and NK cells, the invasive ability of Ishikawa cells was not significantly affected (*all P > 0.05*) (**Supplementary Figure S1B**), suggesting that ITZ inhibits the cytotoxic effects of CD8^+^ T and NK cells. Conversely, the inhibitory effect of ITZ in combination with TAMs on Ishikawa cell invasion was found to be significantly more pronounced than that of ITZ or TAMs administered individually (*all P ≤ 0.001*). This phenomenon was observed across multiple treatment time points (**Figures 2B–D**).

Additionally, TAMs in combination with ICI effectively suppressed Ishikawa cells invasion (all *P ≤ 0.001*) (**Figures 2B–D**). It is notable that the addition of ITZ to the TAMs + ICI combination resulted in a further amplification of the inhibitory effect, which resulted in a significant reduction in invasive activity (*P ≤ 0.001*) (**Figures 2B–D**). These results suggest a synergistic effect of ITZ and ICI in targeting Ishikawa cells invasion, providing a promising therapeutic strategy for the treatment of EC.

### Itraconazole promotes INF-γ and IL-10 secretion and induces polarization of tumor-associated macrophages

**Figure 3:**
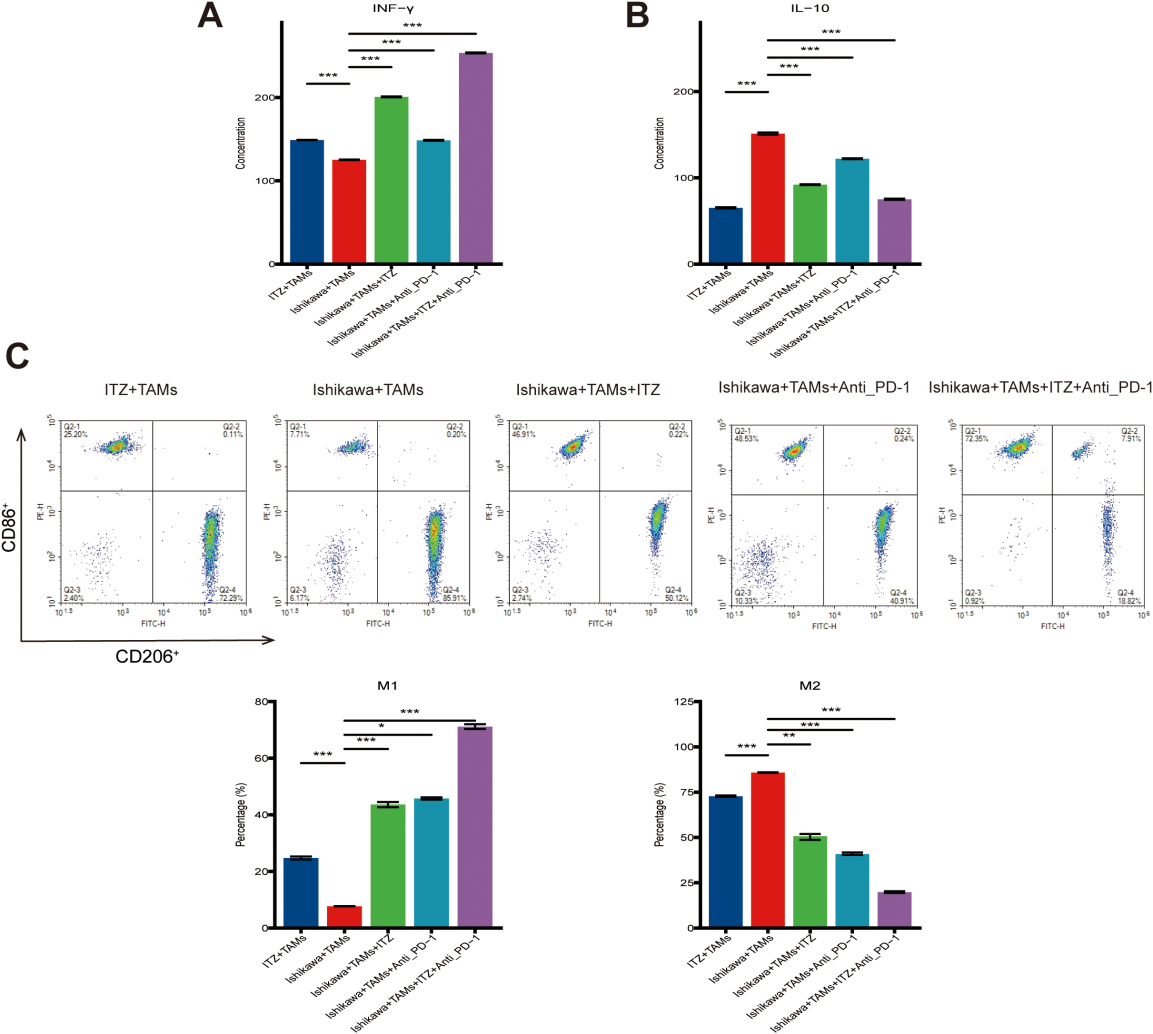
ITZ modulates cytokine secretion and induces the polarization of TAMs. **(A-B)** ELISA analysis of IFN-γ and IL-10 secretion levels in TAM-conditioned media. **(C)** Flow cytometry analysis of TAMs polarization. *P < 0.05, **P ≤ 0.01, ***P ≤ 0.001..

To elucidate the role of TAMs in ITZ-mediated inhibition of Ishikawa cells proliferation and invasion, we analyzed cytokine levels in TAMs-conditioned media using ELISA. Compared to the untreated co-culture system, ITZ treatment significantly increased the concentration of IFN-γ while reducing IL-10 levels in the TAMs-conditioned media (*P ≤ 0.01*) (**Figures 3A, B**). Similar trends were observed in the co-culture system treated with either ITZ or ICI, both of which led to elevated IFN-γ and decreased IL-10 levels (all *P < 0.05*) (**Figures 3A, B**). Notably, the combination of ITZ and ICI maximized these effects, resulting in the highest IFN-γ levels and the lowest IL-10 concentrations (**Figures 3A, B**).

Flow cytometry further revealed that ITZ treatment promoted TAMs polarization towards the M1 phenotype while reducing the proportion of M2-type TAMs. Specifically, the percentage of CD86^+^ M1-type TAMs increased, whereas the percentage of CD206^+^ M2-type TAMs decreased following ITZ treatment (*P ≤ 0.01*) (**Figure 3C**). Similarly, the addition of either ITZ or ICI to the co-culture system enhanced M1 polarization and suppressed M2 polarization (all *P < 0.05*) (**Figure 3C**). Importantly, the combination of ITZ and ICI elicited the most pronounced effects, achieving the greatest increase in M1-type TAMs and reduction in M2-type TAMs (**Figure 3C**).

### Itraconazole inhibits the Wnt/β-catenin signaling pathway in tumor-associated macrophages by downregulating Wnt-3a and β-catenin and upregulating Axin-1

**Figure 4:**
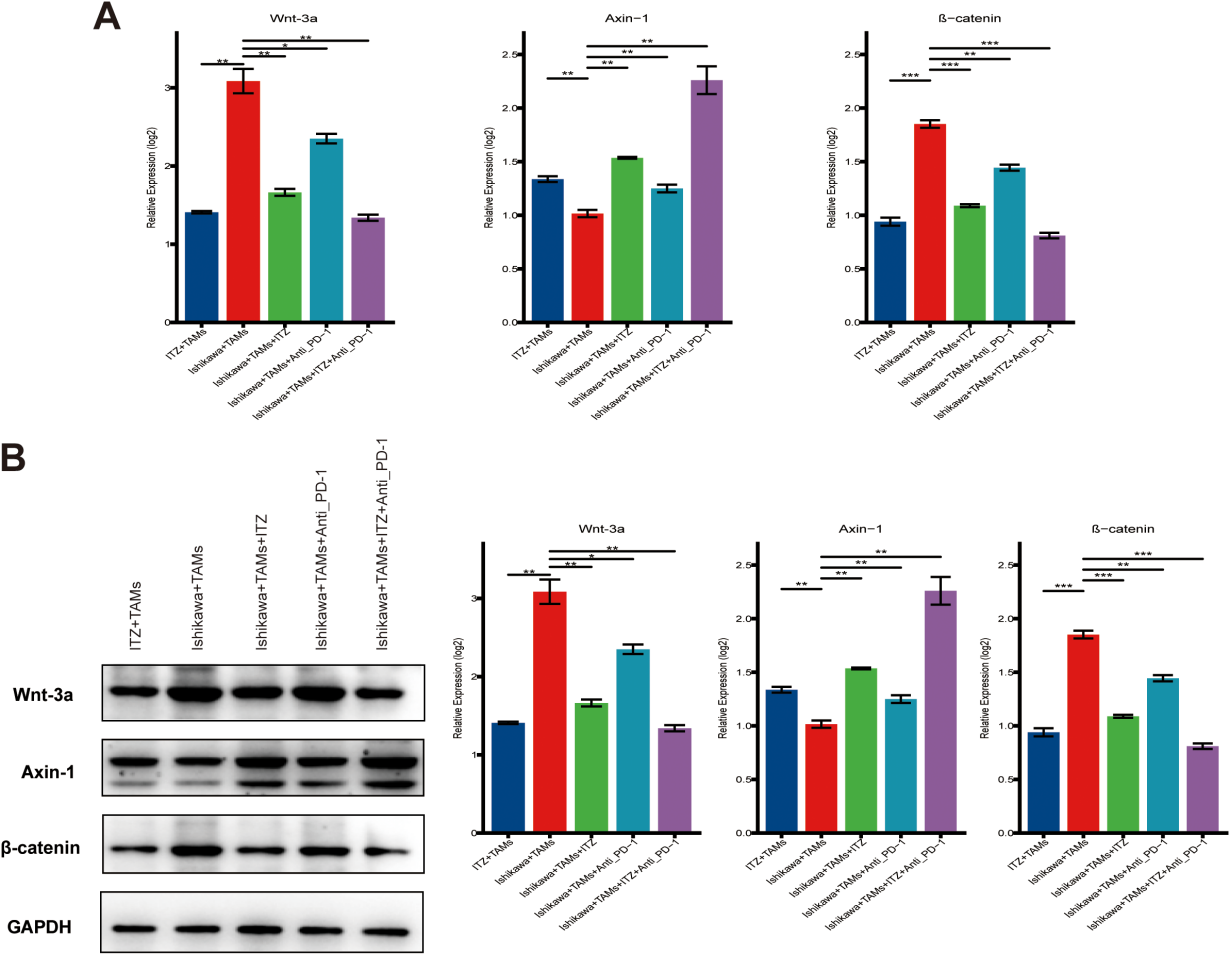
ITZ inhibits the Wnt/β-catenin signaling pathway in TAMs. **(A)** QT-PCR analysis of Wnt/β-catenin pathway-related genes in TAMs treated with ITZ, ICI and their combination. **(B)** Western blotting of Wnt-3a, β-catenin, and Axin-1 protein expression in TAMs treated with ITZ, ICI and their combination. *P < 0.05, **P ≤ 0.01, ***P ≤ 0.001..

To investigate the impact of ITZ on the Wnt/β-catenin signaling pathway in TAMs, we performed RT-PCR and western blotting analyses. RT-PCR results revealed that, compared to the co-culture system, ITZ treatment significantly downregulated the expression of Wnt-3a and β-catenin, while it upregulated Axin-1 expression in TAMs (all *P ≤ 0.01*) (**Figure 4A**). These effects were also observed in the co-culture system treated with either ITZ or ICI, where Wnt-3a and β-catenin levels were decreased and Axin-1 expression was increased (all *P < 0.05*) (**Figure 4A**). Notably, the combination of ITZ and ICI exerted the most significant effects on gene expression, leading to the most pronounced downregulation of Wnt-3a and β-catenin, as well as the highest upregulation of Axin-1 (*P ≤ 0.001*) (**Figure 4A**). Western blotting results further confirmed these findings at the protein level. ITZ treatment significantly decreased the expression of Wnt-3a and β-catenin proteins, while Axin-1 protein expression was markedly upregulated in TAMs compared to the co-culture system (all *P ≤ 0.01*) (**Figure 4B**). Similar trends were observed in the co-culture system treated with either ITZ or ICI, where Wnt-3a and β-catenin proteins were downregulated, and Axin-1 protein expression was upregulated (all *P < 0.05*) (**Figure 4B**). Importantly, the combined treatment of ITZ and ICI resulted in the most significant changes in protein expression, with the greatest reduction in Wnt-3a and β-catenin levels and the highest elevation of Axin-1 protein (*P ≤ 0.001*) (**Figure 4B**).

### Itraconazole inhibits tumor growth and induces tumor-associated macrophages polarization in mice tumor model

**Figure 5:**
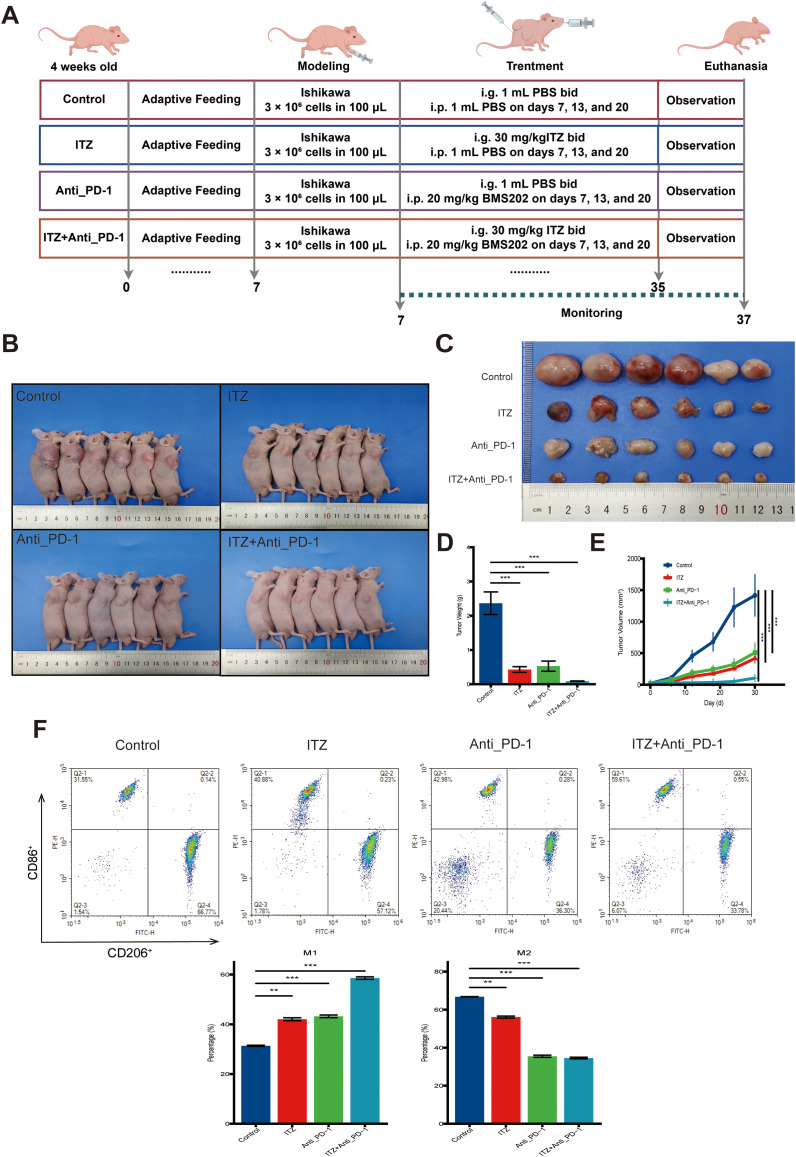
ITZ inhibits tumor growth and induces TAM polarization in a mice tumor model. **(A)** The schematic illustration of the treatment protocol. **(B-C)** Changes in tumor weight and volume in mice treated with ITZ, ICI and their combination. **(D-E)** Quantitative analysis of changes in tumor weight and volume in mice treated with ITZ, ICI and their combination. **(F)** Flow cytometry analysis of TAM polarization in tumor tissues. *P < 0.05, **P ≤ 0.01, ***P ≤ 0.001.

To assess the therapeutic effects of ITZ and ICI *in vivo*, a mice tumor model was established, and tumor weight and volume were measured after treatment (**Figures 5A–C**). Compared to the untreated model group, both ITZ and ICI significantly reduced tumor weight and volume (all *P ≤ 0.001*) (**Figures 5A, C**). Notably, the combination of ITZ and ICI had the most pronounced inhibitory effect on tumor growth (*P ≤ 0.001*), demonstrating a synergistic therapeutic response (**Figures 5D, E**). Additionally, during the treatment period, the activity levels of the mice, their grooming behavior, and overall health remained normal, with no signs of significant toxicity observed in any of the treatment groups.

Further analysis of TAMs phenotypes through flow cytometry revealed significant changes in TAMs polarization. In the untreated tumor model, the proportion of CD206^+^ M2-type TAMs was high, while CD86^+^ M1-type TAMs were relatively low (**Figure 5F**). Following treatment with either ITZ or ICI alone, the proportion of CD86^+^ M1-type TAMs was upregulated, and the proportion of CD206^+^ M2-type TAMs was downregulated (all *P ≤ 0.01*), indicating a shift towards a pro-inflammatory M1-type phenotype (**Figure 5F**). The combination of ITZ and ICI further amplified this effect, leading to the maximal increase in M1-type TAMs and the greatest reduction in M2-type TAMs (*P ≤ 0.001*) (**Figure 5F**).

**Figure 6:**
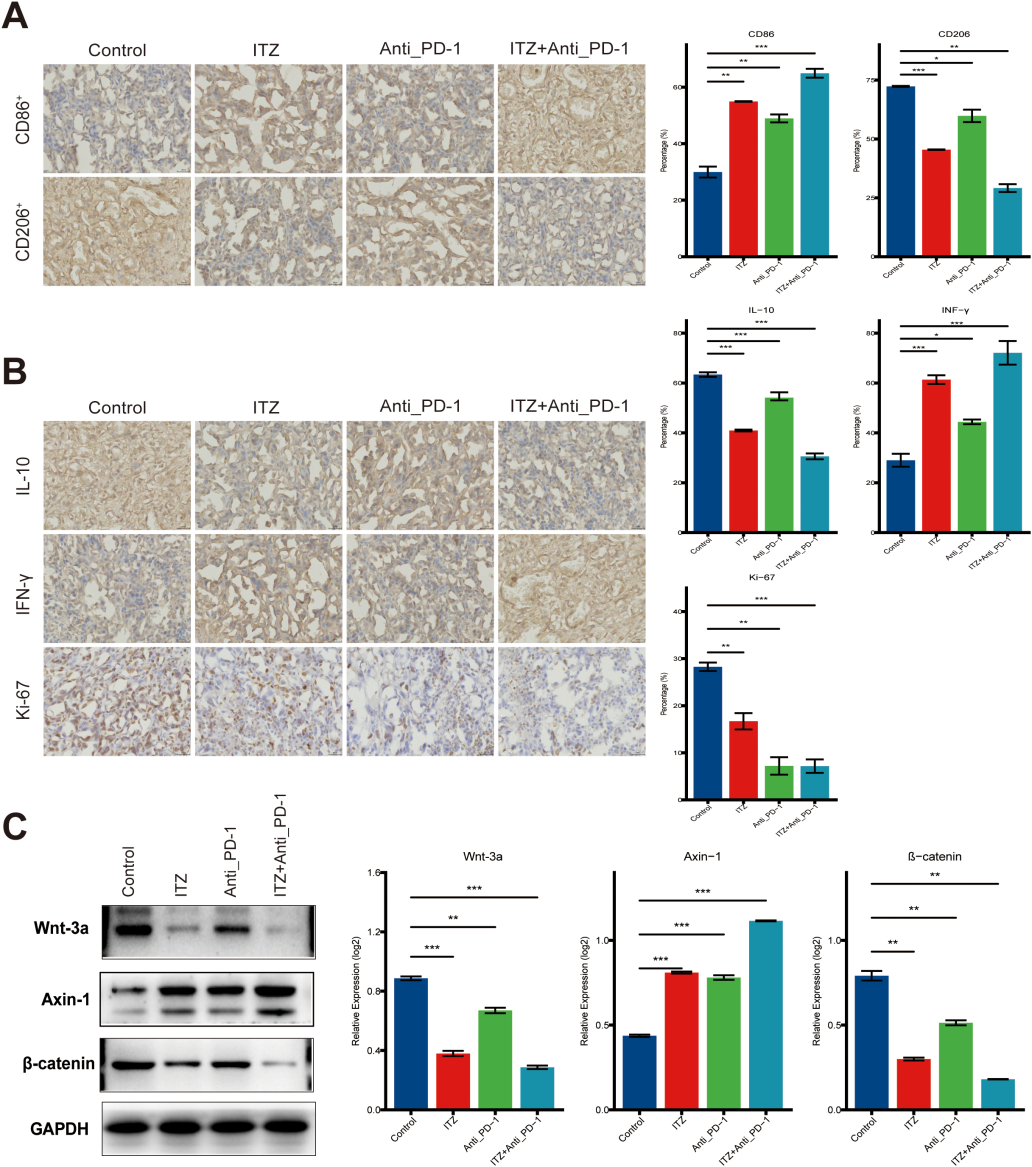
ITZ inhibits the Wnt/β-catenin signaling pathway in a mice tumor model. **(A, B)** Immunohistochemistry analysis of tumor tissues. **(C)** Western blotting of Wnt-3a, β-catenin, and Axin-1 protein expression in TAMs. *P < 0.05, **P ≤ 0.01, ***P ≤ 0.001..

Immunohistochemistry results revealed that, compared to the untreated model group, treatment with ITZ or ICI alone significantly reduced the expression levels of CD206, IL-10, and Ki-67, while the expression of CD86 and INF-γ was significantly increased (all *P < 0.05*) (**Figure 6A, B**). The combination of ITZ and ICI exhibited the most pronounced effects, resulting in the maximal reduction of CD206, IL-10, and Ki-67, as well as the most significant downregulation of CD86 and INF-γ (all *P ≤ 0.01*) (**Figures 6A, B**). In addition, ICI treatment led to a notable augmentation in CD8 and CD56 expression in mouse tumor tissues, in comparison to the untreated control group (all *P ≤ 0.001*) (**Supplementary Figure S1C**). Conversely, both CD8 and CD56 expression were significantly diminished following ITZ monotherapy or ITZ combined with ICI treatment (all *P < 0.05*) (**Supplementary Figures S1C**).

### Itraconazole inhibits the Wnt/β-catenin signaling pathway in a mice tumor model by upregulating Wnt-3a and β-catenin and downregulating Axin-1

To assess the impact of ITZ and ICI on the Wnt/β-catenin signaling pathway in a mice tumor model, we performed western blotting. Compared to the untreated control group, treatment with ITZ or ICI alone led to a significant downregulation of Wnt-3a and β-catenin protein expression, and a significant upregulation of Axin-1 protein (all *P ≤ 0.01*) (**Figure 6C**). The combination treatment of ITZ and ICI resulted in the most significant changes (*P ≤ 0.01*), showing the most pronounced reduction in Wnt-3a and β-catenin, and the greatest increase in Axin-1 expression (**Figure 6C**).

## Discussion

The development of antitumor drugs typically necessitates substantial financial investment and extended development cycles, posing significant challenges for many prospective therapeutic options to achieve market availability. In recent years, drug repurposing (DRP) has gained prominence as a strategic approach that not only substantially reduces costs but also accelerates the time-to-market, thereby offering patients more rapid access to treatment options (26, 27). The primary advantage of DRP lies in the pre-established validation of the safety and pharmacological properties of existing drugs, which significantly mitigates the risks associated with the development of new pharmaceuticals. This approach is particularly promising in the field of oncology, where it provides renewed hope to patients confronting significant unmet medical needs. Recent studies have demonstrated that reevaluating the antitumor potential of non-oncological drugs can lead to the identification of numerous promising drug candidates for clinical applications (28, 29).

ITZ, a frequently employed antifungal medication, has recently been recognized for its potential antitumor properties and has yielded encouraging outcomes in various cancer studies (28). ITZ has been demonstrated to elicit its antitumor effects by inhibiting tumor cells proliferation through multiple signaling pathways, including Wnt/β-catenin, Hedgehog, and PI3K/AKT/mTOR (8–10, 30, 31). In the present study, we verified that ITZ effectively Ishikawa cells proliferation and invasion through apoptosis induction. These findings are consistent with the antitumor effects of the substance previously reported in the literature for various cancers, including lung, liver, ovarian, pancreatic, and prostate cancers, as well as melanoma (8–10, 30, 31). ITZ exerts a dual function, which serves to inhibit tumor cells proliferation, in addition to its regulatory role in the tumor microenvironment, thereby contributing to antitumor activity. In the field of research into head and neck cancer, Lin et al. (32) discovered that ITZ has the capacity to inhibit O-glycosylation in tumor cells. This inhibition effectively promoted the M1 polarization of TAMs. Meanwhile, Pawelec et al. (33) confirmed in a human allogeneic reactive model that ITZ strongly suppressed lymphocyte proliferation and activity. In this study, it was found that ITZ inhibited tumor growth both *in vitro* and *in vivo*. It is important to note that this effect was synergistic with anti-PD-1 therapy, significantly enhancing the efficacy of immunotherapy. Furthermore, it was found that following ITZ treatment, the infiltration abundance of CD8^+^ T and NK cells in mouse tumor tissues was significantly reduced, while their inhibitory effect on Ishikawa cell invasion capacity was significantly weakened. Conversely, the inhibitory effect of ITZ in combination with TAMs on Ishikawa cell invasion was found to be significantly more pronounced than that of ITZ or TAMs administered individually.

It has been established through previous studies that the phenotype of TAMs is a pivotal factor in determining the efficacy of immunotherapy for malignant tumors (12, 34, 35). The activation of the Wnt/β-catenin signaling pathway has been demonstrated to induce the formation of the M2-type in TAMs, thereby weakening the efficacy of immunotherapy. In consideration of ITZ’s regulatory influence on the Wnt/β-catenin signaling pathway, the present study sought to investigate the potential inducible effects of ITZ on TAMs polarization and its capacity to modulate the efficacy of immunotherapy. Our findings demonstrate that, when co-cultured with Ishikawa cells, TAMs predominantly manifest the M2-type, thereby significantly amplifying the invasive capacity of Ishikawa cells. However, following ITZ treatment, TAMs in the co-culture system predominantly shifted to the M1-type, and the invasive capacity of Ishikawa cells was significantly reduced compared to ITZ alone, suggesting that ITZ induces a shift in TAMs from the M2-type to the M1-type. In addition, research has demonstrated that ICI also promote TAMs polarization, thereby inhibiting the invasive capacity of Ishikawa cells, which is consistent with the findings of Gordon et al. (36). Moreover, when ICI were combined with ITZ, the proportion of M1-type TAMs was significantly highest, and the invasive capacity of Ishikawa cells was the weakest, indicating a significant synergistic effect between the two. Consistent with the *in vitro* results, both ITZ and ICI monotherapy significantly reduced tumor weight and volume in mice tumor model and increased the proportion of CD86^+^ M1-type TAMs while reducing CD206^+^ M2-type TAMs. At the same time, the combination of ITZ and ICI exhibited the most pronounced inhibitory effect. In conclusion, ITZ not only inhibits tumor cells proliferation but also reprograms TAMs towards a more pro-inflammatory M1 phenotype, enhancing immunotherapy. The combination of ITZ with ICI shows a synergistic effect, significantly improving tumor growth inhibition both *in vitro* and *in vivo*. These findings highlight the potential of ITZ as an effective adjuvant in immunotherapy of EC patient.

Mechanistically, it was elucidated that ITZ amplifies the antitumor effects of ICI through modulation of the Wnt/β-catenin signaling pathway, thereby inducing the polarization of TAMs. Specifically, ITZ was observed to downregulate the expression of Wnt-3a and β-catenin while upregulating Axin-1 at both the mRNA and protein levels, thereby effectively inhibiting the activation of this pathway in TAMs. *In vivo* studies demonstrated that ITZ effectively promoted the polarization of TAMs towards the M1-type, aligning with *in vitro* findings. Furthermore, ITZ was found to downregulate tumor markers such as Ki-67 and key components of the Wnt/β-catenin signaling pathway, including Wnt-3a and β-catenin, while upregulating Axin-1 expression. ITZ is a multitargeted therapeutic agent. Its ability to modulate both tumor cells proliferation and the tumor microenvironment through the Wnt/β-catenin signaling pathway has significant potential for enhancing the efficacy of immunotherapy. Nevertheless, it is imperative to acknowledge the challenges that must be confronted when attempting to translate these encouraging preclinical findings into viable clinical applications. Firstly, while ITZ demonstrated effective antitumor activity in murine models, the translation of these findings to human patients requires careful evaluation in clinical trials to assess both the efficacy and safety of ITZ in combination with ICIs. The immune system’s differences between humans and mice may lead to variable responses, and thus, further studies are necessary to understand the full scope of ITZ’s effects in human tumor microenvironments. Furthermore, as a multitargeted agent, ITZ may engender off-target effects that could complicate its clinical use, and optimization of dosing regimens is required to maximize its therapeutic potential while minimizing potential adverse effects.

Despite the promising results, this study has some limitations. The results were primarily based on Ishikawa cells, which were selected after preliminary screening due to their superior viability in co-culture systems. However, this may limit the generalizability to other endometrial cancer models. In addition, while our research focused on TAMs due to their critical role in immune regulation, we did not comprehensively evaluate the effects of ITZ on other immune cell populations within the tumor microenvironment, which warrants further investigation. Furthermore, the immunotherapy component of this study used only a single preclinical immune checkpoint inhibitor, BMS202, a small molecule PD-1/PD-L1 inhibitor selected for its stability, low cost and suitability for *in vitro* studies (37). Although BMS202 provides a convenient and sensitive model for early-stage research, it does not fully replicate the complexity of clinically approved ICIs such as pembrolizumab or nivolumab. Future studies using clinically validated agents are needed to better assess the translational potential of the combination strategy presented here.

## Conclusions

In summary, our study underscores the potential of itraconazole as a multitargeted therapeutic agent against endometrial cancer, demonstrating efficacy in inhibiting tumor proliferation, invasion, and progression through both direct tumoricidal effects and immune modulation. The observed synergistic interaction between itraconazole and immune checkpoint inhibitors further highlights its promise in combination therapies, necessitating additional clinical investigations to optimize its application in the treatment of endometrial cancer.

## Data Availability

The original contributions presented in the study are included in the article/**Supplementary Material**. Further inquiries can be directed to the corresponding author.
