# Supplementary material for: Itraconazole inhibits the Wnt/β-catenin signaling pathway to induce tumor-associated macrophages polarization to enhance the efficacy of immunotherapy in endometrial cancer
Source: Front Oncol. 2025 Jul 8;15:1590095. doi: 10.3389/fonc.2025.1590095 (PMC12279481; doi:10.3389/fonc.2025.1590095)
Supplement: Supplementary file 1 [file DataSheet1.docx]

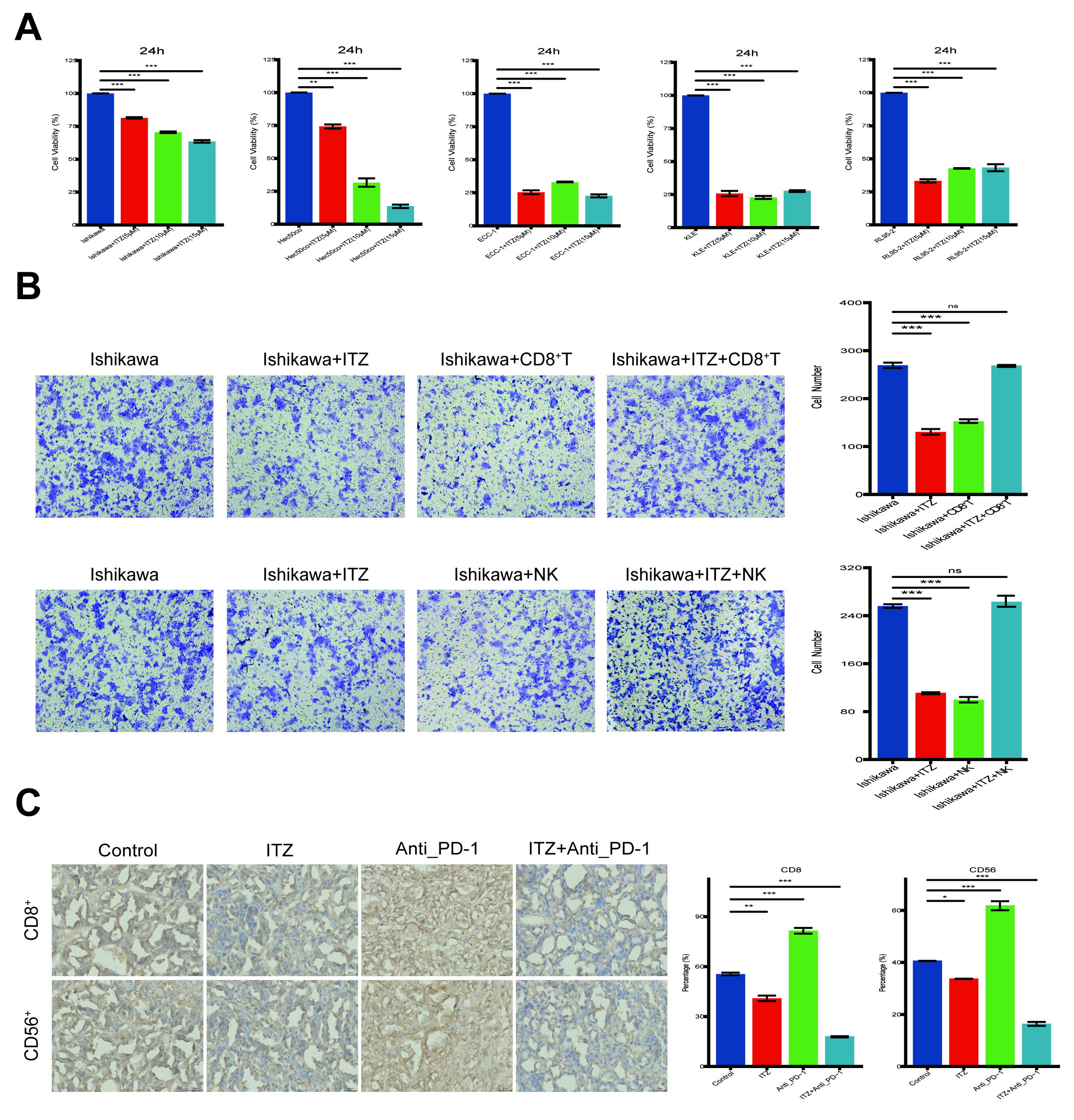


**Supplementary Figure 1.** ITZ inhibits the proliferation of endometrial cancer cells and the cytotoxicity of CD8^+^ T and NK cells. (A) The viability of different endometrial cancer cells treated with increasing concentrations of ITZ (5 µM, 10 µM, and 15 µM) for 24 h was assessed using the CCK-8 assay. (B) Transwell invasion assay showing the invasive ability of Ishikawa cells treated with ITZ, CD8^+^ T and NK cells individually or in combination for 24 h. (C) Immunohistochemistry analysis of tumor tissues. *^ns^P ≥ 0.05, *P < 0.05, **P ≤ 0.01, ***P ≤ 0.001.*
